# Safflower CtFT genes orchestrating flowering time and flavonoid biosynthesis

**DOI:** 10.1186/s12870-024-05943-3

**Published:** 2024-12-23

**Authors:** Zhiling Li, Lili Yu, Abdul Wakeel Umar, Jiaruo Wang, Jian Zhang, Nan Wang, Min Zhang, Na Yao, Naveed Ahmad, Xiuming Liu

**Affiliations:** 1 College of Life Sciences, Engineering Research Center of the Chinese Ministry of Education for Bioreactor and Pharmaceutical Development, Changchun, 130118 China; 2 Institute for Safflower Industry Research of Shihezi University, Pharmacy College of Shihezi University, Key Laborataty of Xinjiang Phytomedicine Resource and Utilization, Ministry of Education, Shihezi, 832003 China; 3 BNU-HKUST Laboratory of Green Innovation, Advanced Institute of Natural Sciences, Beijing Normal University at Zhuhai (BNUZ), Zhuhai, 519087 China; 4 Ginseng and Antler Products Testing Center of the Ministry of Agriculture PRC Jilin Agricultural University, Changchun, 130118 China; 5 Joint Center for Single Cell Biology, Shanghai Collaborative Innovation Center of Agri-Seeds, School of Agriculture and Biology, Shanghai Jiao Tong University, Shanghai, 200240 China

**Keywords:** Safflower, *CtFTs*, Flower formation, Flavonoid biosynthesis

## Abstract

**Background:**

Safflower thrives in dry environments but faces difficulties with flowering in wet and rainy summers. Flavonoids play a role in flower development and can potentially alleviate these challenges. Furthermore, the *FLOWERING LOCUS T* (*FT*) family of *phosphatidylethanolamine-binding protein* (*PEBP*) genes play a crucial role in the photoperiodic flowering pathway. However, their direct impact on flowering and flavonoid biosynthesis under different light duration is elusive.

**Results:**

Utilizing the genome sequencing of Safflower (Jihong NO.1), the current study identifies three specific genes (*CtFT1*, *CtFT2*, and *CtFT3*) that exhibit upregulation in response to long-day conditions. The overexpression of *CtFT2*, displayed an early, whereas *CtFT1* and *CtFT3* late flowering phenotype in *Arabidopsis thaliana*. Interestingly, the transient overexpression of *CtFT1* in safflower leaves caused early flowering, while overexpressing *CtFT2* and *CtFT3* led to late flowering. Additionally, overexpressing *CtFT3* in Arabidopsis and *CtFT1*, *CtFT2*, and *CtFT3* in safflower leaves, significantly increased flavonoid synthesis.

**Conclusions:**

These findings showed that overexpression of *CtFT* genes could affect the flowering time and significantly increase the flavonoid content of safflower. The function of *CtFT* gene is different in safflower and Arabidopsis. This study provides valuable insights into the role of *CtFT* genes in flower formation and flavonoid synthesis in safflower, which may help in improving safflower breeding quality and its adaptability to diverse environmental conditions.

**Supplementary Information:**

The online version contains supplementary material available at 10.1186/s12870-024-05943-3.

## Background

Safflower (*Carthamus tinctorius*) is widely used in Traditional Medicine especially in the treatment of various cardiovascular diseases and gynecological diseases and oil production [1, 2]. Safflower flowers are traditionally used in many countries (e.g., Arabia, China, Egypt, Iran, India, Japan, and Korea) for food, food coloring, health, cosmetics, and cut flowers [3]. Safflower contains a variety of bioactive components such as flavonoids, fatty acids, lignans, and alkaloids. These components contribute to its anti-inflammatory [4], antioxidant [5] and other pharmacological activities [6]. Flavonoids found in safflower are extensively utilized in various medications. Hence, the flavonoid content serves as an important indicator to assess the safflower’s quality as a medicinal plant. Safflower typically thrives in arid climates such as West Asia and China [7]. Safflower planted in the spring takes approximately 3–4 months, whereas safflower sown in autumn requires about 6–8 months to complete its growth cycle [8]. However, in some areas where safflower is sown in spring, the flowering period of safflower often encounters summer rainy days, resulting in late flowering, and flower decay, which is leading to reduced seed yield. Therefore, a method is needed to regulate the flowering of safflower either before or after the rainy season. Molecular marker identification is crucial for modifying safflower to withstand the rainy season while maintaining its normal flowering time. Specific genes related to drought tolerance, waterlogging resistance, and most importantly flowering are targeted using molecular markers. This process accelerates breeding and enhances safflower’s resilience and productivity.

Floral transformation is regulated by various exogenous and endogenous stimuli [8] via six pathways: photoperiod pathway, gibberellin pathway, vernalization pathway, temperature pathway, age pathway, and autonomous pathway [9–11]. These floral pathways are interconnected by several integrated genes, including FLOWERING LOCUS T (FT). FT belongs to the phosphatidylethanolamine binding protein (PEBP) family. PEBP family members: TWIN SISTER OF FT (TSF), TERMINAL FLOWER 1 (TFL1), BROTHER OF FT AND TFL1 (BFT), MOTHER OF FT AND TFL1 (MFT), Arabidopsis thaliana CENTRORADIALIS (ATC) and their homologs have been found in various species. They are involved in the regulation of plant flowering and other growth and development processes [12, 13]. The flowering process in plants involves the activation of *FT* genes, triggered by a transcription factor called CONSTANS (CO) in the leaves [14]. *FT* gene product (FT protein) is produced and transported to the stem apical meristem (SAM), facilitated by the interaction with a protein called FT-INTERACTING PROTEIN1 (FTIP) [15]. In the SAM, the FT protein forms a complex with other proteins, including the 14-3-3 protein and the bZIP transcription factor FLOWERING LOCUS D (FD), which plays a crucial role in the regulation of flowering in plants. This complex, known as the floral activation complex (FAC), promotes the transition of the SAM from vegetative growth to flowering [16]. FAC activates the expression of floral meristem characteristic genes *APETALA 1* (*AP1*), *SUPPRESSOR OF OVEREXPRESSION OF CONSTANS 1* (*SOC1*), and *FRUITFUL* (*FUL*), and controls floral transformation in plants [17].

The overexpression of the *FT* genes causes early flowering in Arabidopsis thaliana, and so far a similar function has been found in other plants [18]. Recent research has illuminated an aspect that the diverse isoforms within the *FT* gene family, revealing that certain variants possess a distinct ability to inhibit their own flowering process. Certain genes, like *GmFT4* in soybeans and *HaFT1* and *HaFT4* in sunflowers, wield contrasting effects on flowering regulation [19]. Beyond their role in flowering, *FT* genes encompass various tasks, for example, the *FT-like* gene in wheat influences seed germination, and in rice, the *Hd3a* gene orchestrates not only flowering but also the intricate process of tiller formation [20, 21]. The increasing range of functions performed by *FT* genes highlights their impressive versatility. While their primary role is in regulating flowering time, these genes may also participate in secondary pathways like flavonoid biosynthesis, thereby diversifying their contributions to plant functions.

Flavonoids are a group of naturally occurring plant compounds that play vital roles in plants, contributing to pigmentation, UV protection, defense against pathogens, and scavenging reactive oxygen species (ROS) [22, 23]. Flavonoids are classified based on their chemical structures, particularly the arrangement of functional groups within the molecules [24, 25]. This classification gives rise to various classes, including flavones, flavonols, flavanones, flavanols (catechins), anthocyanins, isoflavones, chalcones, and aurones [24, 26]. They key enzymes involved during this pathway includes chalcone synthase (CHS), Chalcone isomerase (CHI), Flavanone 3-hydroxylase (F3H), Flavonoid 3’-hydroxylase (F3’H), Flavonol synthase (FLS), Dihydroflavonol 4-reductase (DFR) and Anthocyanidin synthase (ANS). Many transcription factors also regulate flavonoid biosynthesis by regulating key enzyme genes. For example, transcription factors MYB, bHLH and WD40 can form MYB-bHLH-WD40 (MBW) ternary complex. Different types of MYB, bHLH and WD40 proteins can form different MBW complexes and show different functions in the regulation of flavonoid biosynthesis [27]. In addition, bZIP, MADS, ERF, WRKY and other transcription factor families are also involved in the biosynthesis of flavonoids [29–31].

Safflower flavonoids compounds mainly concentrated in the petals, therefore, in the formation of safflower flower must be accompanied by the increase of flavonoids biosynthesis, and *FT* genes as safflower multiple flowering pathway integration gene, we hypothesized that the safflower *FT* genes may be involved in regulating the biosynthesis of flavonoid substances in addition to the regulation of flowering. In this study, three *FT* genes of safflower were identified and isolated. Through heterologous expression in Arabidopsis and transient overexpression of *FT* genes in safflower, the changes of flowering time and phenotype were observed, and the expression changes of related genes during flowering were determined. At the same time, the effect of overexpression of *CtFT* genes on flavonoid content in Arabidopsis and safflower was determined. The expression changes of key enzyme genes in flavonoid metabolism and synthesis were determined. It provides a new view for preliminarily exploring the regulation mechanism of safflower flowering and cultivating safflower varieties with high flavonoid content.

## Results

### Identification and physicochemical properties of CtFTs from the PEBP gene family in safflower

The safflower genome was analyzed using HMMER and SMART software tools. After removing redundant sequences, a total of 7 *CtPEBP* genes were identified with the following accessions: CCG016893.1, CCG011803.1, CCG016894.1, CCG013216.1, CCG019669.1, CCG000764.1, and CCG027220.1. Among these genes, three belong to the *FT* gene family and were named *CtFT1* (CCG016893.1), *CtFT2* (CCG011803.1), and *CtFT3* (CCG016894.1) based on their similarity to homologous genes in Arabidopsis. The *CtFT* genes vary in sequence length ranging from 525 bp (*CtFT2*), 528 bp (*CtFT1*), and 531 bp (*CtFT3*). These sequences encode proteins of different lengths: 174 amino acids for CtFT2, 175 amino acids for CtFT1, and 176 amino acids for CtFT3. In terms of molecular weight, CtFT2 has a weight of 19.68 kDa, CtFT1 has a weight of 19.71 kDa, and CtFT3 has a weight of 19.93 kDa. The isoelectric points of these proteins also vary, with CtFT2 at 6.29, CtFT1 at 7.75, and CtFT3 at 6.83 (Table S1). All members of this protein family display negative GRAVY values, indicating their hydrophobic nature. Subcellular localization predictions suggest that CtFT3 is localized in the cytoplasm, while the other two members are predicted to be located in the nucleus.

### Chromosomal distribution and phylogenetic analysis

**Fig. 1:**
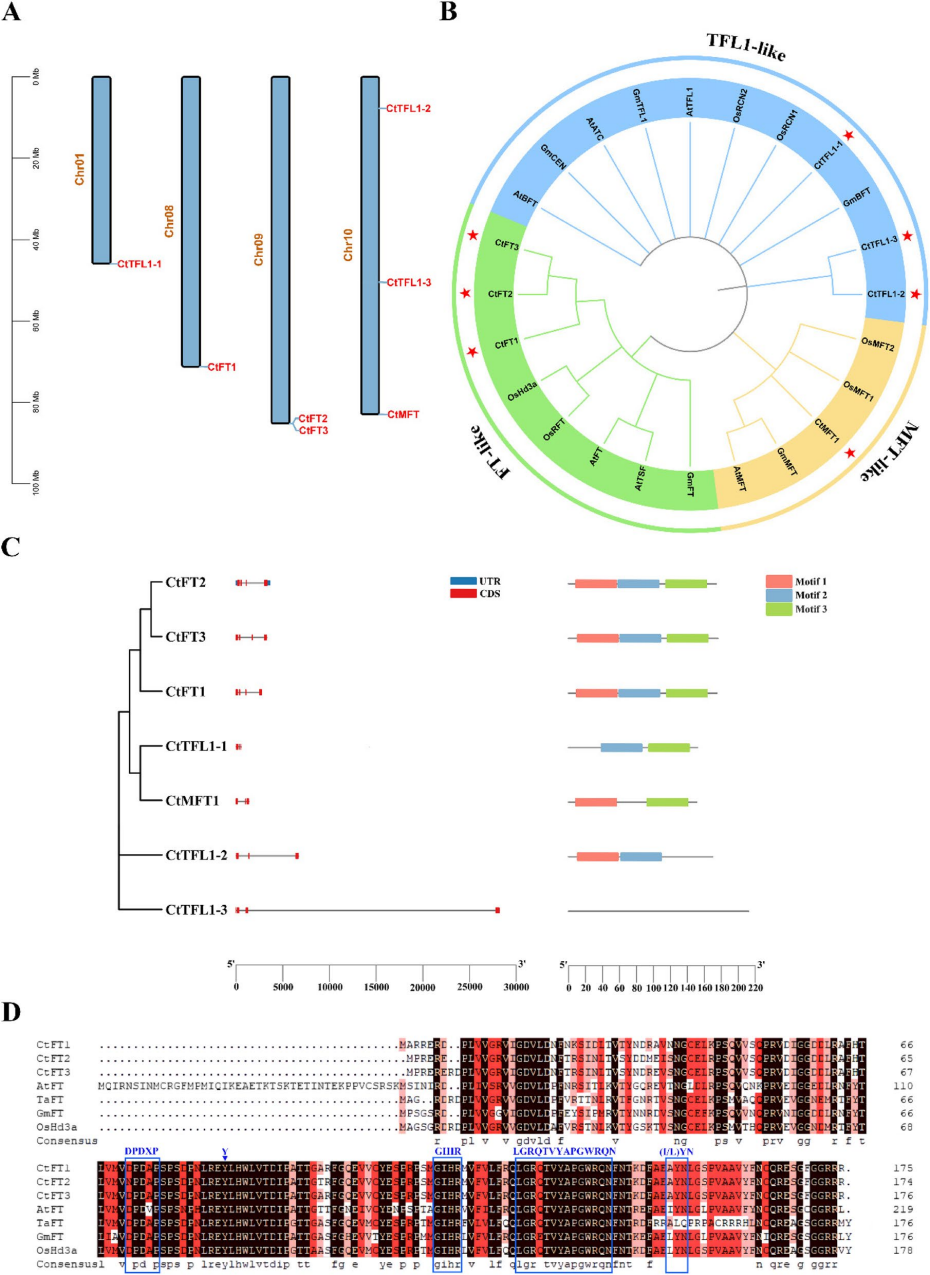
Chromosomal localization, Phylogenetic analysis and conserved topology of PEBP family in safflower. **A** the chromosome wise distribution of CtPEBP genes in safflower. The blue vertical lines indicate different safflower chromosome (Chr) and the number of chromosomes is indicated at the left side of each chromosome. The left scale bar used for locating genes was demonstrated in Mb. **B** Phylogenetic analysis of PEBP family members from safflower, Arabidopsis, rice and soybean. The five-pointed star represents safflower PEBP family members. **C** The gene structure and conserved protein motif analysis of PEBP family genes in safflower. The blue colored boxes represent untranslated regions (UTR) and red colored boxes represents coding sequences (CDS). The connections between UTR and CDS lines represent introns. The scale at the bottom is drawn to facilitate the comparison of the relative lengths of various genes and proteins. **D** Multiple sequence alignment of CtFT and their homologs and identification of conserved domains of PEBP family

To understand the distribution patterns and gene duplication of *CtPEBP* genes, we utilized the genomic annotation information of safflower to determine their chromosomal locations. As shown in Fig. 1A, the 7 *CtPEBP* genes are unevenly distributed across 4 different safflower chromosomes. Notably, chromosome 10 contains the highest number of three genes (*CtFL1-2*, *CtFL1-3*, and *CtMFT*). Chromosomes 9 contain 2 genes (*CtFT2* and *CtFT3*), while chromosomes 1 and 8 each house one *CtPEBP* gene (*CtFL1-1*) and (*CtFT1*), respectively. Furthermore, to investigate the evolutionary relationships of CtPEBP protein members with their homologs in other species, we generated a phylogenetic tree using the neighbor-joining method in MEGA software. This analysis was based on the full-length amino acid sequences of PEBPs obtained from safflower, Arabidopsis, rice, and soybean. The resulting phylogenetic tree classified the PEBP proteins from different plant species into three subfamilies including FT-like, TFL1-like, and MFT-like (Fig. 1B). Within the CtPEBP protein family, both the FT-like and TFL1-like subfamilies exhibited the clustering of three members in each group, while the MFT-like subgroup contained one member. The clustering of CtFT members with other members of the same family from Arabidopsis, rice, and soybean suggests their close evolutionary relationship.

### Gene structure, cis elements, motifs and conserved domain analysis

To gain a more comprehensive understanding of the *CtPEBP* gene family, we utilized GSDS webserver for an in-depth analysis of *CtPEBP* gene structures. As depicted in Fig. 1C, the *CtPEBP* gene family exhibited varying numbers of introns, typically ranging from 2 to 3. Notably, within this family, the FT subfamily displayed the highest intron count, reaching a maximum of 3. Similarly, the number of exons observed in *CtPEBP* genes extended to 4 (Fig. 1C). Furthermore, it is noteworthy that other subfamily members demonstrated consistent patterns in terms of intron and exon numbers, suggesting potential associations between genetic structure and specific biological functions, regulatory pathways, or evolutionary relationships.

Similarly, to provide further insights regarding the potential roles and expression regulatory mechanisms of the *CtPEBP* genes, the PlantCARE website was employed to investigate the cis-acting elements located within the *CtPEBP* genes promoter region (upstream 2 kb region). The results showed the conservation of diverse cis-acting elements, encompassing elements associated with light responsiveness (such as G-box, Box-4, and TCT-motif), elements responsive to plant hormones, stress-related elements, and elements related to growth and development processes (Fig. S1). Notably, the most abundant among these were the plant hormone-responsive elements, which included gibberellin-responsive elements (TATC-box, P-box, and GARE motif), auxin-responsive elements (TGA-element and AuxRR core), salicylic acid-responsive elements (TCA-element), abscisic acid-responsive elements (ABRE), and the CGTCA and TGACG motifs associated with methyl jasmonate (MeJA) responses.

In addition, the MEME online webtool was used to forecast the conserved motifs occur within the CtPEBP family. As illustrated in Fig. 1C, the number of conserved motifs in each CtPEBP protein ranges from Motif 1 to Motif 3. All members of CtFT proteins include all three motifs. On the contrary, CtTFL1-1 contains motif 2 and motif 3, CtMFT1 contains motif 1 and motif 3, and CtTFL1-2 contains motif 1 and motif 2. Noticeably, no motif was detected for CtTFL1-3. The presence of all three motifs in CtFT proteins demonstrates a notable degree of conservation among its constituents. Meanwhile, multiple sequence alignment of CtFTs and other homologs from different species was carried out to identify their conserved domains (Fig. 1D). The analysis unveiled that, except a notable change in CtFT2, where the initial aspartic acid (D) was substituted with asparagine (N) within the conserved D-P-D-x-P domain, CtFT proteins possess well-conserved domains of the PEBP family. It is well established that PEBP family include D-P-D-x-P, G-x-H-R, and the crucial tyrosine (Y) at the 85th amino acid position distinguishing them from the TFL1 subfamily. Notably, at position 144, a key amino acid residue, glutamine (Q), resides within a conserved domain denoted as Segment B (LGRQTVYAPGWRQN), which plays a regulatory role in floral formation. Furthermore, it is noteworthy that safflower distinguishes itself from other species by having alanine (A) as the first amino acid in the YN triad. This intriguing variation suggests the possibility that CtFTs might possess functions distinct from those of their analogous proteins in other species .

### Potential role of CtFTs in flowering under altered photoperiods

**Fig. 2:**
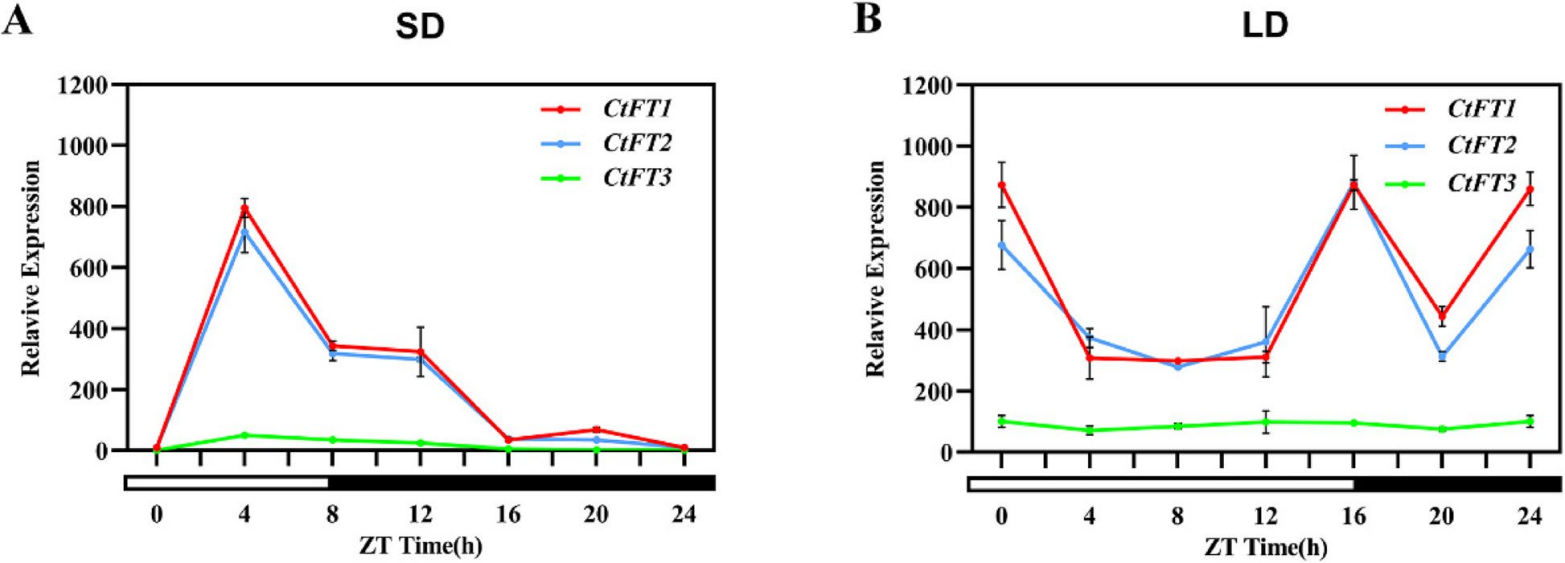
Expression patterns of *CtFT* genes in safflower under contrasting light conditions Expression pattern of safflower *CtFT* genes (**A**) under short-day conditions (8 h light / 16 h darkness). **B** under long-day conditions (16 h light / 8 h darkness)

To investigate the potential role of *CtFTs* in the regulation of flowering under altered photoperiod conditions, batches of safflower plants with uniform growth conditions were exposed to altered photoperiods, including long day (LD) and short day (SD), while keeping other environmental conditions constant. The expression patterns of three key *CtFT* genes (*CtFT1*, *CtFT2*, and *CtFT3*) within a 24-h time frame were closely monitored.Under the short-day conditions (SD), we observed distinct expression patterns for the *CtFT* genes. Noticeably, *CtFT1* and *CtFT2* displayed an obvious increase in expression at 4 h of light, followed by a subsequent decline at 12 and 24 h, forming an approximate 8-h oscillatory pattern (Fig. 2B). In contrast, *CtFT3* exhibited consistently low expression levels throughout the entire observation period under the SD condition (Fig. 2A). Conversely, under long day conditions (LD), *CtFT1* and *CtFT2* exhibited distinct expression profiles. Their highest expression levels were observed at 16, and 24 h of light, with a relative decrease during the 4 to 12-h interval. Notably, *CtFT3* maintained a consistently low level of expression throughout the entire observation period under the LD condition (Fig. 2B). Interestingly, when comparing the two photoperiod conditions, the expression levels of *CtFTs* were consistently higher under LD conditions in comparison to SD conditions. Additionally, the expression patterns of *CtFT1* and *CtFT2* were remarkably synchronized under both LD and SD conditions, suggesting their pivotal roles in photoperiod-dependent flowering regulation. In contrast, *CtFT3* displayed a stable and consistently lower expression level throughout the observation period under both photoperiod conditions. These findings indicate that *CtFT* genes may play a crucial role in mediating the safflower response to altered photoperiods, thus influencing the timing of flowering. The consistent and synchronized expression of *CtFT1* and *CtFT2* under both LD and SD conditions speculates at their potential role in the core regulation of flowering in safflower.

### CtFT genes likely regulate flowering time in transgenic Arabidopsis

**Fig. 3:**
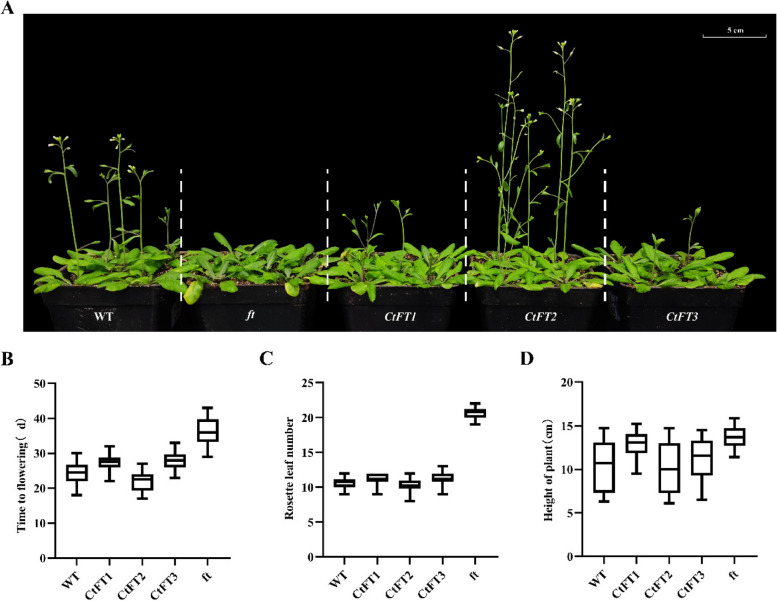
Phenotypic analysis of *CtFTs* transgenic Arabidopsis compared to WT and *ft* mutants. **A** Representative T3 transgenic Arabidopsis lines alongside WT (Col-0) and *ft* mutants were cultivated under long-day (LD) conditions and photographed simultaneously five weeks after planting. **B** Time of flowering (days). **C** The number of rosette leaves at the time of blossoming, measured from plants with four leaves of uniform size at transplantation. (D) The length of the main stem at the onset of flowering in Arabidopsis. Each dataset is based on 20 biological replicates

To comprehensively assess the role of *CtFT* genes in regulating flowering time, transgenic Arabidopsis including mutant and overexpression lines were generated using agrobacterium mediated transformation method (Fig. S2 and S3). Two high-expression lines in T3 population for each *CtFT* genotype (Fig. S4) were screened and selected for analyzing phenotypic traits in combination with mutant and WT lines. The phenotypic analysis, involving comparisons between *CtFTs* transgenic Arabidopsis, wild-type (WT), and *ft* mutants, revealed distinct flowering time outcomes. Notably, *CtFT2* lines exhibited an early flowering phenotype, whereas *CtFT1* and *CtFT3* lines displayed late flowering characteristics (Fig. 3A), consistent with the statistical data on flowering time (Fig. 3B). Additionally, the number of rosette leaves in *CtFT2* transgenic lines was slightly lower than in WT Arabidopsis, indicating a reduction in the vegetative growth period. In contrast, Arabidopsis thaliana overexpressing *CtFT1* and *CtFT3* exhibited a slightly higher number of rosette leaves compared to the WT. Remarkably, the *ft* mutants showed a significantly higher number of rosette leaves compared to the other four lines (Fig. 3C). Furthermore, the length of the main stem of Arabidopsis with *CtFT2* overexpression was slightly shorter than that of the WT at the onset of flowering, whereas Arabidopsis overexpressing *CtFT1* and *CtFT3* displayed longer main stems compared to WT and *ft* mutant lines (Fig. 3D). Together, our findings suggest that the overexpression of the *CtFT2* gene leads to shorten the vegetative growth period of Arabidopsis, resulting in decrease number of rosette leaves and a shorter main stem in *CtFT2* lines. Conversely, *CtFT1* and *CtFT3* overexpression appears to have the opposite effect, delaying flowering and promoting vegetative growth. These results provide valuable insights into the distinct roles of *CtFT* genes in regulating the timing of flowering in transgenic Arabidopsis.

### Transient overexpression of CtFT1 induced early flowering in safflower

**Fig. 4:**
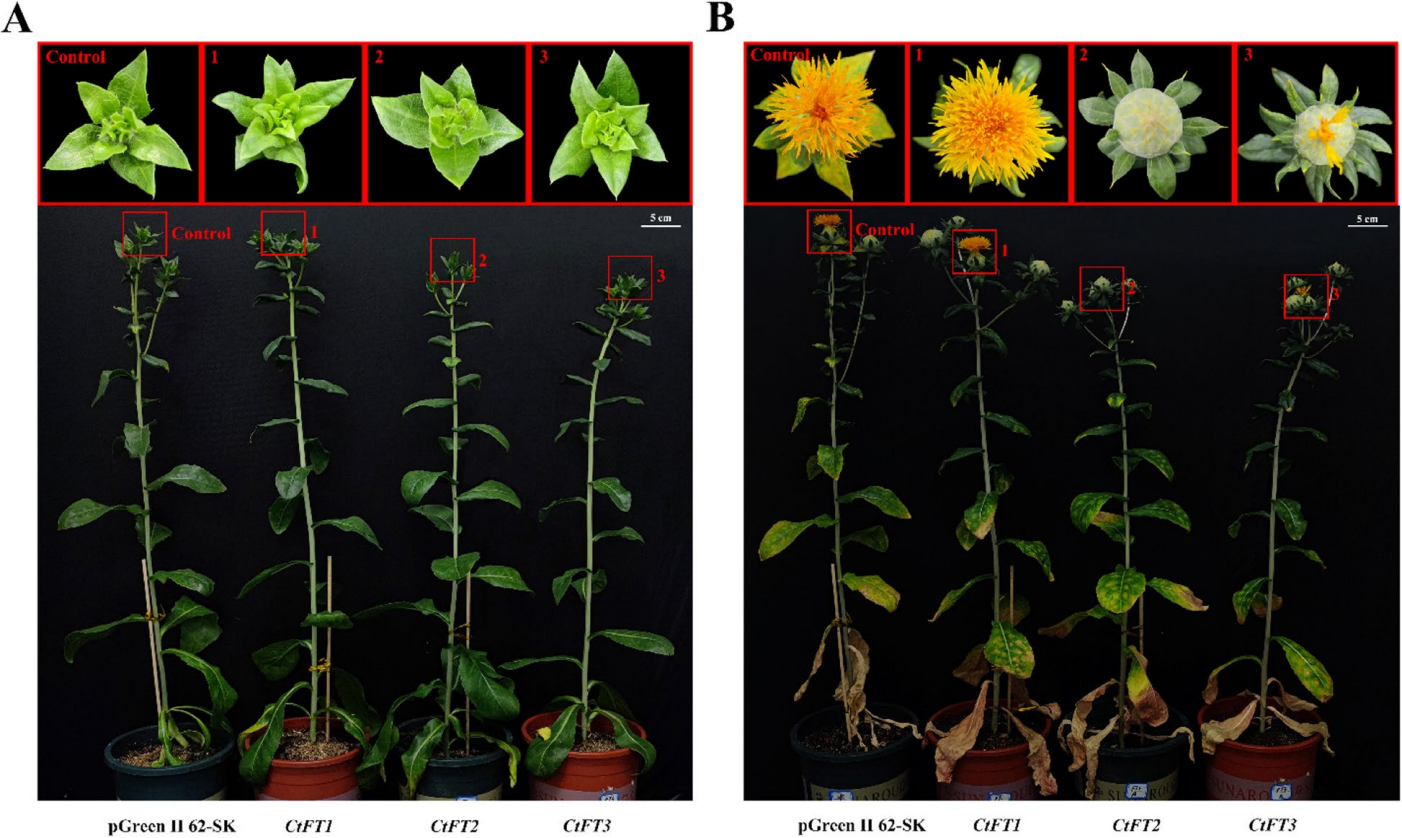
Differential regulation of flowering time in safflower after transient overexpression of *CtFT* genes. **A** Prior to injection of pGreenII62-SK-CtFTs and empty vectors. **B** After 20 days of injection with pGreenII62-SK-CtFTs and empty vector, illustrating variation in flowering time

To further confirm the putative role of *CtFT* genes in regulating flowering time in safflower, we employed a transient overexpression approach. For this purpose, the pGeenII62-SK-CtFTs expression vector (Fig. S5) was constructed and transiently expressed in safflower. The results obtained 20 days after injection demonstrated distinct flowering responses in safflower following the transient overexpression of *CtFT* genes. Interestingly, safflower plants with transient overexpression of *CtFT1* exhibited early flowering, eventually progressing to the full flowering stage. In contrast, the control group injected with the pGeenII62-SK empty vector experienced a delay in flowering, with only the appearance of tubular petals at the early flowering stage. Moreover, safflower plants with transient overexpression of *CtFT2* remained in the bud stage, indicating a significant delay in flowering, whereas *CtFT3* overexpression reached to the early flowering stage, although with a reduced number of tubular petals compared to the control group( Fig. 4A and B). These observations indicate that the overexpression of *CtFT1* in safflower significantly promoted early flowering, while both *CtFT3* and *CtFT2* induced delayed flowering to varying degrees. However, *CtFT2* appeared to delay flowering to a greater extent than *CtFT3* in two separate biological repeat groups (Figs. S6 and S7). These findings highlight the pivotal role of *CtFT* genes in modulating the timing of flowering in safflower, with *CtFT1* promoting early flowering, *CtFT2* causing a substantial delay, and *CtFT3* inducing a somewhat delayed flowering, albeit with fewer tubular petals.

### Overexpression of CtFT genes enhanced flavonoid accumulation by inducing the expression of key flavonoid pathway genes in Arabidopsis

**Fig. 5:**
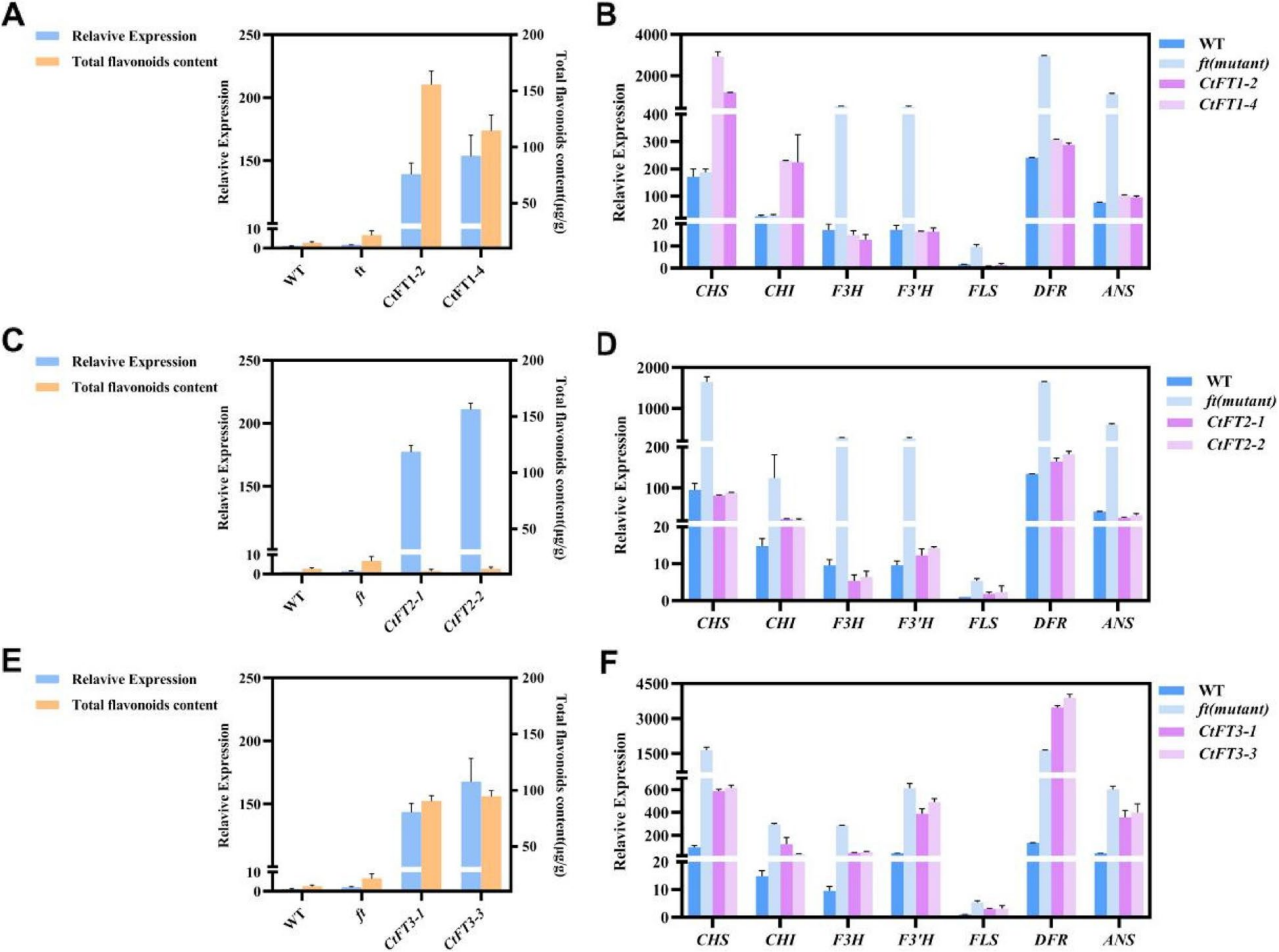
Correlation of *CtFT* genes and flavonoid accumulation in Arabidopsis. **A**, **C**, **E**: Gene expression and total flavonoid content analysis in WT, *ft* mutant, and two OE transgenic lines of *CtFT1 CtFT2* and *CtFT3* genes. **B**, **D**, **F**: Relative expression level of key genes involved in flavonoid biosynthesis pathway in WT, *ft* mutant, and two OE transgenic lines of *CtFT1*,* CtFT2* and *CtFT3* genes. Each value represents three biological repeats, and three technical repeats. The error line represents the standard deviation

To validate the correlation between *CtFT* genes and flavonoid accumulation, we conducted comprehensive analyses of *CtFT* gene expression and total flavonoid content in Arabidopsis, including WT, *ft* mutant, and two overexpressing (OE) transgenic lines for each *CtFT* gene. Our results, presented in Fig. 5, reveal distinctive patterns of gene expression and flavonoid content in the CtFT-transgenic lines when compared to the WT and *ft* mutant lines. Interestingly, CtFT-transgenic lines exhibited significantly upregulated expression of their respective *CtFT* genes relative to the WT and *ft* mutant lines. However, the accumulation of total flavonoids displayed a nuanced variation among these transgenic lines. Notably, *CtFT1* and *CtFT3* OE transgenic lines exhibited elevated expression of their respective *CtFT* genes, which correlated with increased flavonoid accumulation compared to the WT and mutant lines (Fig. 5A and C). In contrast, *CtFT2* OE lines demonstrated a moderate increase in *CtFT2* gene expression, whereas the total flavonoid content remained higher in the WT and mutant lines (Fig. 5B). These findings suggested important insights into the crucial role of *CtFT* gene expression and flavonoid accumulation, highlighting potential regulatory dynamics for each *CtFT* gene in Arabidopsis.

Furthermore, the relative expression levels of genes encoding key enzymes in the flavonoid biosynthesis pathway were also examined in WT, *ft* mutant, and CtFT-transgenic lines. In *CtFT1* transgenic lines, the expression of most flavonoid biosynthetic genes, except *CHS* and *CHI*, was significantly downregulated in the OE transgenic lines compared to the *ft* mutant line. However, two exceptions were observed - *DFR* and *ANS*, which exhibited higher expression in the OE transgenic lines than in the WT. The higher expression of *CHS* and *CHI* in *CtFT1* OE transgenic lines well aligned with the higher flavonoid accumulation in these lines, implying that *CtFT1* may play a pivotal role in the upstream regulatory pathway of flavonoid biosynthesis. In *CtFT2* transgenic lines, the selected genes showed reduced expression compared to the *ft* mutant lines. However, some genes, including *CHI*, *F3’H*, *DFR*, and *FLS*, displayed higher expression levels in *CtFT2* transgenic lines compared to the WT. In contrast, *F3H* exhibited lower expression in *CtFT2* transgenic lines when compared to the WT. These results suggest that *CtFT2* may not significantly impact the expression of key flavonoid pathway genes and their role in flavonoid accumulation. In the case of *CtFT3* transgenic lines, two genes located in the downstream pathway of flavonoids, *DFR* and *ANS*, displayed higher expression in the OE transgenic lines compared to the WT and *ft* mutant lines. Other genes exhibited reduced expression compared to the *ft* mutant lines but increased expression compared to the WT. These findings suggest that *CtFT3* might associates with the accumulation of flavonoid by influencing the downstream regulatory pathway of flavonoids. These findings collectively underscore the multifaceted regulatory roles of *CtFT* genes in the complex network of flavonoid biosynthesis, shedding light on their significance in regulating flavonoid accumulation in Arabidopsis.

### Transient overexpression of CtFT genes also promotes flavonoids accumulation by regulating the expression of key flavonoid pathway genes in safflower

**Fig. 6:**
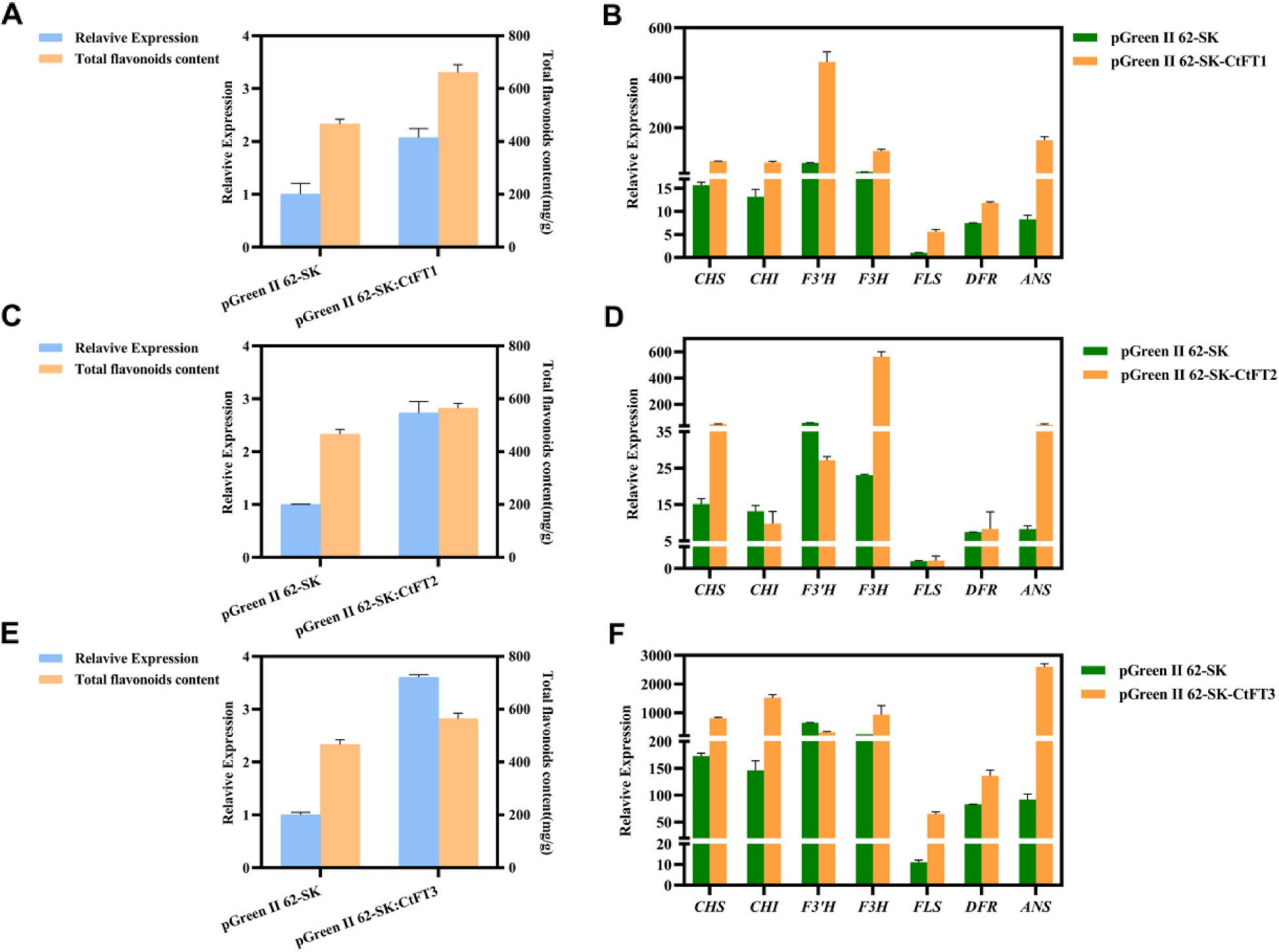
Transient overexpression of *CtFT* genes expression of *FT* gene, content of total flavonoids and expression of key enzyme genes in flavonoid biosynthesis pathway in safflower and empty vector control. **A**, **C**, **E**: relative expression and total flavonoid content of *CtFT* genes in instantaneous overexpression of safflower and injection empty vector safflower. **B**, **D**, **F**: relative expression of key enzyme genes of flavonoid biosynthesis pathway in instantaneous overexpression of safflower and injection empty vector safflower. Each value represents three biological repeats, and each creature repeats three technical repeats. The error line represents the standard deviation of the repetition of three organisms

To further confirm whether *CtFT* genes impart flavonoid accumulation in safflower, we investigated the *CtFT* genes expression and flavonoid accumulation in safflower. For this purpose, the transiently OE safflower lines containing pGreenII62-SK-CtFTs recombinant plasmid and control lines containing pGreenII62-SK empty vector were used. The results showed that CtFT-transgenic safflower line exhibited significantly enhanced expression of their respective *CtFT* genes compared to control lines. Simultaneously, the accumulation of total flavonoids content was also detected suugested higher accumulation in OE plants than that in control plants(Fig. 6 ACE). Furthermore, we also explored the impact of transient overexpression of *CtFT1*, *CtFT2*, and *CtFT3* on the expression of key flavonoid biosynthetic pathway genes in safflower. The results revealed that all the genes were upregulated in *CtFT1* transgenic lines, with *F3’H*, *ANS*, and *F3H* showing the most significant increases (Fig. 6B). Similarly, most of the genes were abundantly expressed in the transient overexpression lines of *CtFT2* gene except except *CHI* and *F3’H* (Fig. 6D). Similar findings were also observed in transient overexpression lines of *CtFT3*, indicating up-regulation of the most of the genes except *F3’H* (Fig. 6F). To sum up, transient overexpression of *CtFT* genes in safflower leaves enhances the synthesis of flavonoids in safflower leaves (Fig. 6 BDF). the findings from the transient overexpression of *CtFT* genes in safflower not only provide compelling evidence of their pivotal role in augmenting flavonoid biosynthesis but also illuminate their potential as valuable targets for enhancing flavonoid production, which has promising implications for the pharmaceutical, nutraceutical, and agricultural industries.

## Materials and methods

### Plant materials and growth conditions

The safflower seeds of the “Jihong No.1” commercial cultivar were procured by the Engineering Research Center of Bioreactor and Pharmaceutical Development at Jilin Agricultural University. These seeds underwent a rigorous process of sterilization and stratification, after which safflower seedlings were cultivated under controlled conditions. For the long-day treatment, a photoperiod of 16 h of light and 8 h of darkness was maintained at a constant temperature of 25°C over a span of 28 days in a specialized growth room equipped with precise environmental controls. To study the gene expression patterns, samples were meticulously collected on the 28th day after germination at multiple time points, including 0, 4, 8, 16, and 24 h post-germination. All Arabidopsis lines, on the other hand, were grown separately in a growth chamber under a consistent photoperiod of 16 h of light and 8 h of darkness, at a controlled temperature of 22°C, until they reached the seedling stage. T-DNA-inserted mutant lines of Arabidopsis were sourced from the Arashare Arabidopsis mutant library, while the wild-type Arabidopsis Columbia-0 (col-0), was acquired from the Engineering Research Center of Bioreactor and Pharmaceutical Development at Jilin Agricultural University. Each experimental group and its corresponding control group were subjected to a rigorous experimental design, involving three biological replicates to ensure the robustness and reliability of the research findings.

### Identification and characterization of FT genes from the PEBP gene family in safflower

The most recent version of safflower genome was obtained from The Genome Database of Carthamus tinctorius (http://safflower.scuec.edu.cn). For the identification of *CtPEBP* genes, we obtained the PBP domain HMM model (PF01161) from the Pfam database (https://pfam.xfam.org). Subsequently, we employed HMMER 3.1 software to scour genome protein databases, applying an e-value threshold of 1.0 × e^−5^. The genes that were obtained from the aforementioned methods were categorized as potential members of the *PEBP* gene family. Subsequently, we subjected them to validation using SMART, Pfam, and the CDD database to ensure the integrity and completeness of the PEBP domain. Any redundant sequences or those featuring an incomplete PEBP domain were excluded from subsequent analyses. Furthermore, the GFF3 files containing essential information regarding the genomic locations of safflower genes were also obtained from the aforesaid database. Then, by using the versatile TB tools visualization software, we performed the chromosomal localization of genes belonging to the *PEBP* family in safflower. Moreover, we constructed a phylogenetic tree for the PEBP family by employing the Neighbor-Joining (NJ) method within MEGAX. The initial alignment was performed using ClustalW, and the Jones-Taylor-Thornton (JTT) model was subsequently chosen as the most appropriate substitution model. To account for variation among sites, the Gamma Distributed (G) rates were incorporated. Finally, we assessed the reliability of the branches through 1000 Bootstrap replicates.We utilized the iTOL online website (https://itol.embl.de/itol.cgi) to refine the evolutionary tree. The amino acid sequences were aligned using DNAMAN. Gene structure visualization was accomplished through GSDS 2.0 (http://gsds.gao-lab.org/). Additionally, we employed MEME (https://meme-suite.org/meme/tools/meme) for further analysis.

### Expression analysis of CtFT genes under altered photoperiods

To investigate the transcriptional regulation of *CtFT* genes in safflower under different light conditions including short day (8 h light / 16 h darkness) and long day (16 h light / 8 h darkness), qRT-PCR was conducted. For this purpose, total RNA extraction was carried out using the Tsingke Biotechnology TrellefTMRNAprep Pure Plant Kit (China). Then, first-strand cDNA synthesis was carried out with the MonScriptTMRTIII All-In-One Mix along with dsDnase (Monad, JiangSu, China). Primers for each *CtFT* gene sequence were designed with Primer 3 software and then synthesized by Sangon Biotech (ChangChun, China) platform. Real-time quantitative PCR was conducted in the Mx3000p Multiplex Quantitative PCR System (Agilent, California, USA) using MonAmpTMChemoHS qPCR Mix purchased from (Monad, Jiangsu, China). The PCR thermal cycle included an initial step of 10 min at 95℃, followed by 40 cycles of 10 s at 95℃ and 30 s at 60℃. The 18 S ribosomal RNA genes was used as an internal control. To calculate the relative gene expression, the 2-delta CT method was employed. Each experiment was conducted with at least three independent biological replicates, and two technical repeats were performed in each case.

### Acquisition of CtFT-transgenic Arabidopsis thaliana via Agrobacterium-mediated transformation

To validate the function of *CtFT* genes in vivo, we cloned the *CtFT* genes, employing Takara LA Taq DNA polymerase, using cDNA derived from safflower leaves as the template. The heat and shock method was used to transform the target fragment of *CtFT* genes using Dh5α competent cells. The transformed cells were then incorporated into pEASY-T1 cloning vector, and then allowed to cultivate on on LB agar plates supplemented with kanamycin. Subsequently, individual colonies were transferred to liquid LB medium containing kanamycin. The plasmids were then extracted and the results confirmed through Sanger sequencing. Next, the homology arm primers were designed witht the help of Primer 3Plus (https://www.bioinformatics.nl/cgi-bin/primer3plus/primer3plus.cgi). Using the cloning vector of the *CtFT* gene as a template, *CtFT* genes were cloned and ligated into pCAMBIA3301 plant expression vector using the Monad (China) single-segment seamless cloning kit (MC40201). Then, the pCAMBIA3301-CtFTs recombinant plasmid was transformed into Agrobacterium EHA105 competent cells and were allowed to grow on YEP liquid medium until OD600 reached at 0.8. Floral dip transformation of wild type A. thaliana was conducted following the instruction given by [28]. Transgenic plants were screened by PCR amplification using Taq DNA polymerase (Promega Corp, Madison, WI, USA) and the corresponding *CtFT* primer pairs.

### Transient overexpression of CtFT genes in safflower

In order to overexpress the *CtFT* gene in safflower, the full-length coding sequence of three *CtFT* genes were inserted into the pGreenII62-SK vector and subsequently transformed into EHA105 Agrobacterium tumefaciens competent cells. The bacterial colonies of A. tumefaciens harboring pGreenII62-SK-CtFTs were cultured until it reached an optical density (OD_600_) of 0.6 in a YEP liquid medium. Following centrifugation, a solution containing 1% acetosyringone (AS) was re-suspended to an OD_600_ of 0.8. This solution was then injected into the abisal side of the safflower leaves after a 3-h incubation period in the dark. The plants were subsequently kept in darkness for 24 h before being placed under normal LD (light-dark) conditions for further cultivation.

### Quantitative analysis of total flavonoid content in transgenic Arabidopsis and safflower

We collected petals from fully blooming flowers of wild-type (WT) and ft mutant Arabidopsis lines, as well as from CtFTs overexpressing transgenic Arabidopsis lines and transiently CtFTs overexpressing safflower lines, to determine the total flavonoid content. The plant tissues were ground into a fine powder using liquid nitrogen and then transferred into test tubes. Approximately, 0.1 g of each plant tissue was used for quantification. Subsequently, 500 µL of methanol and 100 µL of an 8% sodium nitrite solution were added to the test tubes, and the contents were thoroughly dissolved for 5 min. Following this, 100 µL of a 15% aluminum nitrate solution was added, and the mixture was allowed to stand for 5 min. Then, 1 mL of a 6% sodium hydroxide solution was introduced, followed by the addition of 6 mL of distilled water. The mixture was vigorously mixed by vortex oscillation and left undisturbed for 3 min. Finally, the supernatant was carefully transferred into a 2-mL centrifuge tube and centrifuged at 13,000 rpm for 10 min. The supernatant was subjected to ultrasonic cleaning at 50°C for 30 min. Subsequently, the solution was filtered through a 0.22 μm filter, and the quantification of the total flavonoid content was determined by measuring the absorbance spectra in the range of 300–500 nm using a UV-Vis spectrophotometer.

### Expression profiling of CtFT and other key flavonoid pathway genes in transgenic Arabidopsis and safflower

To further explore the *CtFT* transcript abundance in Arabidopsis and safflower transgenic plants, the fresh leaves and petals samples from WT, mutant (*ft*), *CtFTs* overexpressed transgenic Arabidopsis lines and transiently *CtFTs* overexpressed transgenic safflower lines were collected for RNA extraction following the previous protocol of [29] with slight modifications. Then, cDNA templates were synthesized and qRT-PCR analysis was conducted for each experimental group, respectively. First, the relative expression level of *CtFT* in overexpressed transgenic Arabidopsis lines in comparison to WT and mutant lines were conducted using gene specific primers. Similarly, the transcript abundance of *CtFT* in transiently overexpressed transgenic safflower lines in comparison with mock lines was investigated. At the same time, under the same conditions, the qRT-PCR assay was also investigated in each experimental group from both Arabidopsis and Safflower to determine the transcription levels of the seven core structural genes of the flavonoid pathway. Reaction were performed on Real-time PCR system (Mx3000p Multiplex Quantitative PCR System) (Agilent, California, USA) using MonAmpTMChemoHS qPCR Mix purchased from (Monad, Jiangsu, China). A 20 µL reaction mixture including 10 µL SYBR Premix Ex Taq (Tli RNaseH Plus), 0.3 µL ROX Reference Dye, 0.4 µL F/R primer, 1 µL template and 7.9 µL ddH2O was used. The PCR thermal cycle included an initial step of 10 min at 95℃, followed by 40 cycles of 10 s at 95℃ and 30 s at 60℃. The 18 S ribosomal RNA genes was used as an internal control. The expression level was calculated according to the 2−△△Ct method. The primers details used for the qRT-PCR analysis of key structural genes of the flavonoid pathway listed are given in supplementary Tables S2 and S3.

## Discussion

### Floral differentiation of CtFT genes in *Carthamus tinctorius*

Artificially controlling the vegetative growth time of crops to adapt to the climatic environment of different regions or directed accumulation of metabolites, and the cultivation of crop strains with different flowering time has always been the focus of research. As a key gene and integron in plant flowering pathway, *FT* gene plays an important role in the cultivation of crop lines with different flowering time. In this study, based on the genome sequencing of safflower “Jihong No.1”, three *FT* genes of safflower were cloned, and two plants highly expressing *CtFT* gene were obtained. The flowering time of *CtFT2* transgenic Arabidopsis lines was earlier than that of wild type control, and the flowering time of plants with transient overexpression of *CtFT1* in safflower leaves was earlier than that of empty vector control, indicating that *CtFT2* can promote early flowering in Arabidopsis while *CtFT1* can promote early flowering in safflower, which is consistent with the function of *FT* gene in promoting flowering in other species [30, 31]. The overexpression of *CtFT1* and *CtFT2* in Arabidopsis and safflower leads to opposite flowering phenotype, which is rarely reported. It is speculated that it may be due to the species difference between safflower and Arabidopsis, different regulatory networks of floral pathway and different protein composition and conformation related to floral formation.

With the further study of *FT* gene and other genes related to floral pathway, more and more studies have found that the function of *FT* gene differentiates in different species, and *FT* gene can inhibit floral formation in some plants. LenaHarig found that *NtFT1*, *NtFT2* and *NtFT3* are late flowering genes in tobacco, and only *NtFT4* is early flowering gene [32]. Through ectopic expression of longan *DlFTs* gene in Arabidopsis, it was found that *DIFT1* can promote flower formation, while *DlFT2* can inhibit flower formation [33]. Consisitent with this, safflower *CtFTs* also showed similar functional differentiation. We found that the flowering time of *CtFT1* and *CtFT3* transgenic Arabidopsis was delayed than that of WT Arabidopsis, and the down-regulation of *AP1*, *FUL*, *SOC1* and *LFY* genes downstream of *FT* in *CtFT1* and *CtFT3* Arabidopsis was down-regulated compared with WT Arabidopsis. After transient overexpression of *CtFT* genes in safflower, it was found that the flowering time of transient overexpression of *CtFT2* and *CtFT3* in safflower leaves was later than that of empty vector control. To sum up, it is suggested that the heterologous expression of *CtFT1* and *CtFT3* in Arabidopsis is a floral inhibitory gene, while in safflower, *CtFT2* and *CtFT3* are floral inhibitory genes.

However, it is worth noting that from the results of flowering time in Arabidopsis, the late flowering degree of Arabidopsis overexpressing *CtFT1* and *CtFT3* was significantly lower than that of *ft* mutants, but only slightly later than that of WT Arabidopsis, and the expression of downstream floral genes in Arabidopsis was lower than that of WT Arabidopsis but higher than that of *ft* mutants, suggesting that *CtFT1* and *CtFT3* overexpressed in Arabidopsis may not be major flowering inhibitory genes, or only play a limited auxiliary role in the inhibition of flowering. The regulatory network of plant floral formation is huge and complex. The specific effect of *CtFT* genes on plant floral formation and its molecular mechanism still need to be further explored.

### CtFT genes promotes flavonoids biosynthesis in Arabidopsis and safflower

The current study explored the influence of *CtFT* genes on flavonoid biosynthesis in Arabidopsis and safflower, shedding light on potential regulatory mechanisms. While previous research has not directly implicated the *FT* gene in flavonoid regulation, studies on flower formation-related genes have demonstrated their impact on flavonoid content. For instance, the overexpression of the kiwifruit *SVP3* gene was found to inhibit anthocyanin biosynthesis in petals [34], possibly through a pathway akin to the one involving *FT* genes in safflower, as evidenced by our results showing that *CtFT* gene overexpression up regulates key enzymes in the anthocyanin biosynthesis pathway. Similarly, it was reported that the overexpression of *miR172* in stable transgenic apple and Arabidopsis plants led to a reduction in red coloration and induced changes in the levels of flavonoid compounds. These observed phenotypic modifications aligned with the shifts in expression patterns of genes within the anthocyanin biosynthesis branch and other branches of the flavonoid biosynthesis pathways [35]. Consistent with their findings, we also found that CtFT-transgenic Arabidopsis lines exhibited distinct patterns of gene expression and flavonoid content. Notably, *CtFT1* and *CtFT3* overexpression correlated with elevated flavonoid accumulation, suggesting a gene-specific influence of *CtFTs* on flavonoid biosynthesis. Further analysis revealed that *CtFT1* may play a pivotal role in the upstream regulatory pathway, impacting the expression of key flavonoid biosynthetic genes such as *DFR* and *ANS*. *CtFT2*, on the other hand, exhibited a less pronounced impact, with only moderate increases in gene expression observed. In the case of *CtFT3*, downstream pathway genes (*DFR* and *ANS*) showed higher expression, indicating a potential role in modulating later stages of flavonoid biosynthesis. Transient overexpression of *CtFTs* in safflower further confirmed their relevance to flavonoid accumulation, as evidenced by significantly enhanced expression of *CtFT* genes and increased total flavonoid content in CtFT-transgenic safflower lines compared to controls. This effect was consistent across all three *CtFT* genes, supporting their role in promoting flavonoid biosynthesis in safflower leaves. Prior studies have illustrated that in Chrysanthemum morifolium, the ray florets undergo a progressive color transformation from yellow to white throughout flower development [36]. This shift is attributed to an augmentation in flavone and flavonol content, coupled with a potential decline in anthocyanin accumulation. In summary, our findings provide compelling evidence for the involvement of *CtFT* genes in enhancing flavonoid biosynthesis in both Arabidopsis and safflower. The observed gene-specific effects underscore the complexity of the regulatory network governing flavonoid production. These results contribute valuable insights into potential targets for enhancing flavonoid production, with implications for pharmaceutical, nutraceutical, and agricultural applications. Additionally, our findings align with previous studies on flower formation-related genes and their impact on flavonoid content, suggesting a broader connection between floral regulatory pathways and secondary metabolite biosynthesis. Further research is warranted to elucidate the specific mechanisms underlying *CtFT* gene regulation of flavonoid biosynthesis and its potential applications in safflower genetic transformation.

At present, it has not been reported that *FT* gene directly regulates flavonoid biosynthesis, but there have been studies on the effect of flower formation-related genes on flavonoid content. WuRongMei et al. found that overexpression of kiwifruit *SVP3* gene inhibited anthocyanin biosynthesis in petals [34], while other studies showed that *SVP* gene could inhibit the transcription of *FT* gene in flowering pathway [37, 38]. It is speculated that the pathway of kiwifruit *SVP3* inhibiting anthocyanin biosynthesis may be similar to that of *FTs* gene in safflower promoting anthocyanin biosynthesis, which is consistent with the results that the overexpression of *CtFT* gene up-regulates the key enzyme genes of anthocyanin biosynthesis pathway in safflower leaves and Arabidopsis. In this study, Arabidopsis lines with overexpression of *CtFT3* showed late flowering phenotype, and the content of total flavonoids was higher than that of WT, but lower than that of *ft* mutants; Arabidopsis lines with overexpression of *CtFT1* also showed late flowering phenotype, but the total flavonoids content did not increase. Transient overexpression of *CtFT* genes in safflower produced two different phenotypes of early flower and late flower, but the content of total flavonoids increased consistently. It is speculated that the pathway of *CtFT3* promoting flavonoid biosynthesis in Arabidopsis and *CtFTs* promoting flavonoid biosynthesis in safflower leaves may be independent of floral formation, and the specific regulation mechanism needs to be further studied in the stable genetic transformation of safflower.

In flowering plants, the content of flavonoids is usually the highest in petals [39]. Whether exogenous or endogenous factors stimulate plant floral formation, floral organ development can only begin through the integration of genes such as *FT*, *AP1*, *FUL*, *SOC1* and *LFY* [40]. Therefore, it is speculated that floral pathway integration genes may be involved in the regulation of flavonoid biosynthesis in the process of plant floral organ formation. In previous extensive studies, *FT* gene mainly expressed and synthesized *FT* protein in plant leaves, which was transported to plant stem tip for a long distance to regulate flowering [41, 42]. In our previous findings, it was found that the relative expression of *CtFT* genes in petals of safflower at different flowering stages increased continuously from early flowering stage, reached the highest at full flowering stage, and decreased continuously with petal decay. It is consistent with the changing trend of safflower yellow pigment content, which suggests that *CtFTs* can not only receive floral signals from upstream genes in leaves to stem tips to promote flowering, but also may be related to the regulation of flavonoid biosynthesis in petals.

## Conclusion

In this study, we found that *CtFT1* advanced the flowering time of safflower, but delayed the flowering time of Arabidopsis. *CtFT2* delayed the flowering time of safflower but advanced the flowering time of Arabidopsis. *CtFT3* delayed flowering time in both safflower and Arabidopsis. Overexpression of *CtFTs* significantly increased the content of flavonoids in safflower. Overexpression of *CtFT1* and *CtFT3* in Arabidopsis also led to the same result, but the case of *CtFT2* was the opposite. The function of *CtFT* genes in safflower is different from that in Arabidopsis.

## Supplementary Information

Supplementary Material 1.

## Data Availability

All data generated or analyzed during this study are included in the published article and supplementary files. The datasets used and/or analyzed during the current study are available from the corresponding author on reasonable request.
