# Supplementary material for: Safflower CtFT genes orchestrating flowering time and flavonoid biosynthesis
Source: BMC Plant Biol. 2024 Dec 23;24:1232. doi: 10.1186/s12870-024-05943-3 (PMC11665109; doi:10.1186/s12870-024-05943-3)
Supplement: Supplementary file 1 — Supplementary Material 1. [file 12870_2024_5943_MOESM1_ESM.pdf]

Table S1. Physicochemical Properties of Carthamus tinctorius PEBP Family proteins

Gene Name	Gene ID	Protein Length	PI	MW (Da)	Subcellular Localization	Instability Index	GRAVY
CtFT1	CCG016893.1	175	7.75	19719.28	cytoplasm	42.40	-0.406
CtFT2	CCG011803.1	174	6.29	19681.24	cytoplasm	50.17	-0.384
CtFT3	CCG016894.1	176	6.83	19933.46	cytoplasm	45.07	-0.449
CtTFL1-1	CCG013216.1	152	9.16	17259.85	cytoplasm	43.06	-0.297
CtTFL1-2	CCG019669.1	170	4.76	19524.06	microbody	21.35	-0.204
CtTFL1-3	CCG000764.1	212	5.21	23811.78	microbody	58.35	-0.557
CtMFT	CCG027220.1	151	9.55	16987.44	cytoplasm	35.33	-0.384

Table S2. Primers used in qPCR for key structural genes of the flavonoid pathway in Arabidopsis.

Primer Name	Primer Sequence
qRT-AtCHS-F;	AGAAGTTCAAGCGCATGTGC;
qRT-AtCHS-R	AGAGAAGGAGCCATGTAAGCAC
qRT-AtCHI-F;	ACGCCGTTCCTTCTCTATCTG;
qRT-AtCHI-R	ACGGCAGTTTCATTGTCACC
qRT-AtF3H-F;	TCAGATCGTTGAGGCTTGTG;
qRT-AtF3H-R	ATGTCGAAACGGAGCTTGTC
qRT-AtF3'H-F;	TGGATTACAAGCCACACGTG;
qRT-AtF3'H-R	ACGACAAACGCTCCTTCAAC
qRT-AtFLS-F;	TGCAGTGCATGTGAAGAAGC;
qRT-AtFLS-R	CGAGACCTTCTTTCAACGCATC
qRT-AtDFR-F;	TCGGTCCATTCATCACAACG;
qRT-AtDFR-R	TGAGCGTTGCATAAGTCGTC
qRT-AtANS-F;	TGCAAACGATCAAGCCACTG;
qRT-AtANS-R	TTGTCCACTCGCGTTGTTAG
qRT-18srRNA-F;	GAGAAACGGCTACCACATCCAA;
qRT-18srRNA-R	TCGTTTGAGCCCGGTATTGTTA

Table S3. Primers used in qPCR for key structural genes of the flavonoid pathway in Carthamus tinctorius.

Primer Name	Primer Sequence
qRT-CtCHS1-F;	AGACACAAGCGCTCGACATGT;
qRT-CtCHS1-R	ATCTTGGACCAGGTCGAGGAA
qRT-CtCHI1-F;	CGGTATGCAACATGCCGAA;
qRT-CtCHI1-R	AAGATACTTGGCAATGGTTGCG
qRT-CtF3H-F;	GTCAAGTTGCATTCCCTTCTCG;
qRT-CtF3H-R	TTTTGCGGCTGAGATGTGAG
qRT-CtF3'H3-F;	TCCTTAACCCTAGGCTCATGCC;
qRT-CtF3'H3-R	TCTTGCCAGGAGGTGAAAAGC
qRT-CtFLS1-F;	TGAAACAAGTGGTCCACCCAC;
qRT-CtFLS1-R	ACCATAGCCAAGCCTGCAAA
qRT-CtDFR2-F;	GAATCATCGGTTCTAACGGTGG;
qRT-CtDFR2-R	GTTTGTGTAACTGGAGGCCGAG
qRT-CtANS1-F;	TGCCGGTGAAGGAGAAGAAA;
qRT-CtANS1-R	CAGCCCAATCAAGCTTTTGC
qRT-18srRNA-F;	GAGAAACGGCTACCACATCCAA;
qRT-18srRNA-R	TCGTTTGAGCCCGGTATTGTTA

Figure S1

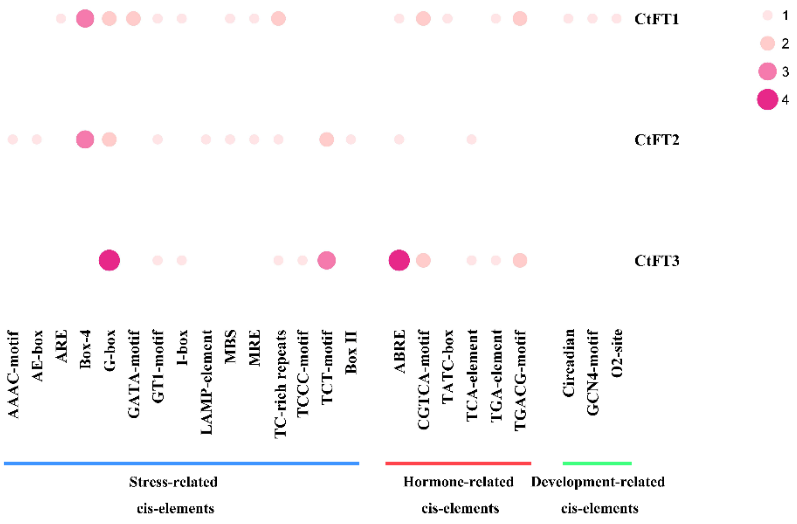

Promoter analysis of *CtFTs* genes.

Figure S2.

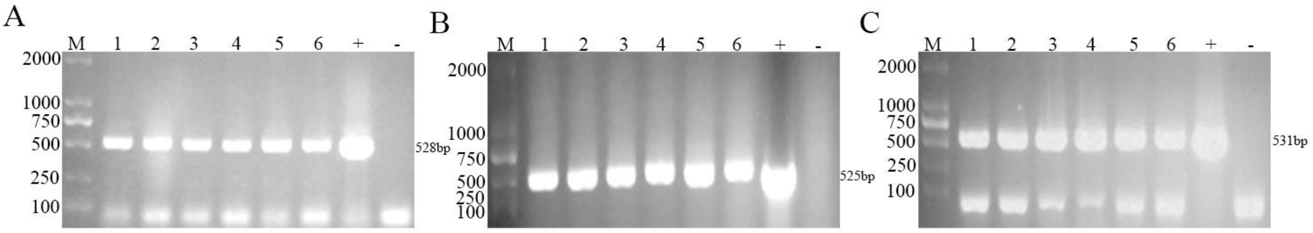

PCR electrophoresis results of E.coli transformed by pCAMBIA3301-CtFTs.
A:CtFT1,B:CtFT2,C:CtFT3

Figure S3

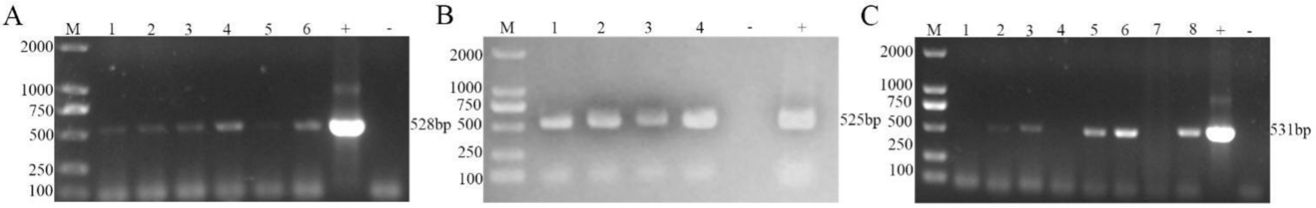

PCR electrophoresis results of Agrobacterium tumefaciens transformed by pCAMBIA3301-CtFTs.
A:CtFT1;**B**:CtFT2;**C**:CtFT3

Figure S4

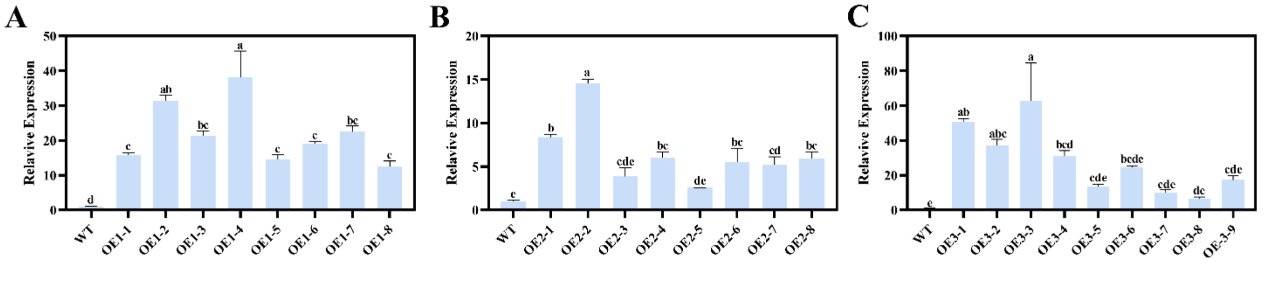

Screening of transgenic *Arabidopsis thaliana* lines with high expression of *CtFTs*
A:*CtFT1* line;**B**:*CtFT2* line;**C**:*CtFT3* line

Figure S5

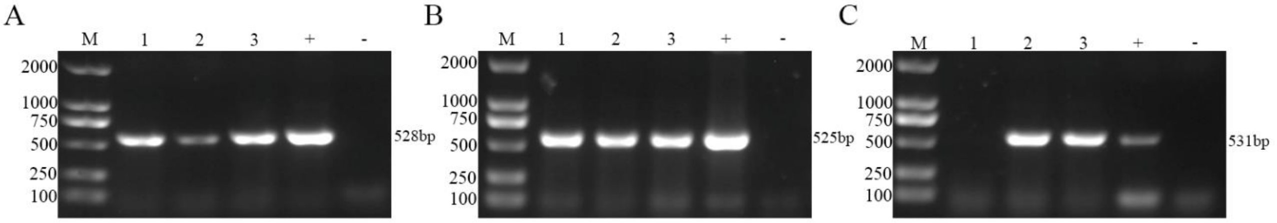

PCR Electrophoresis results of E.coli liquid transformed by pGreen II 62-SK-CtFTs
A:CtFT1;**B**:CtFT2;**C**:CtFT3

Figure S6.

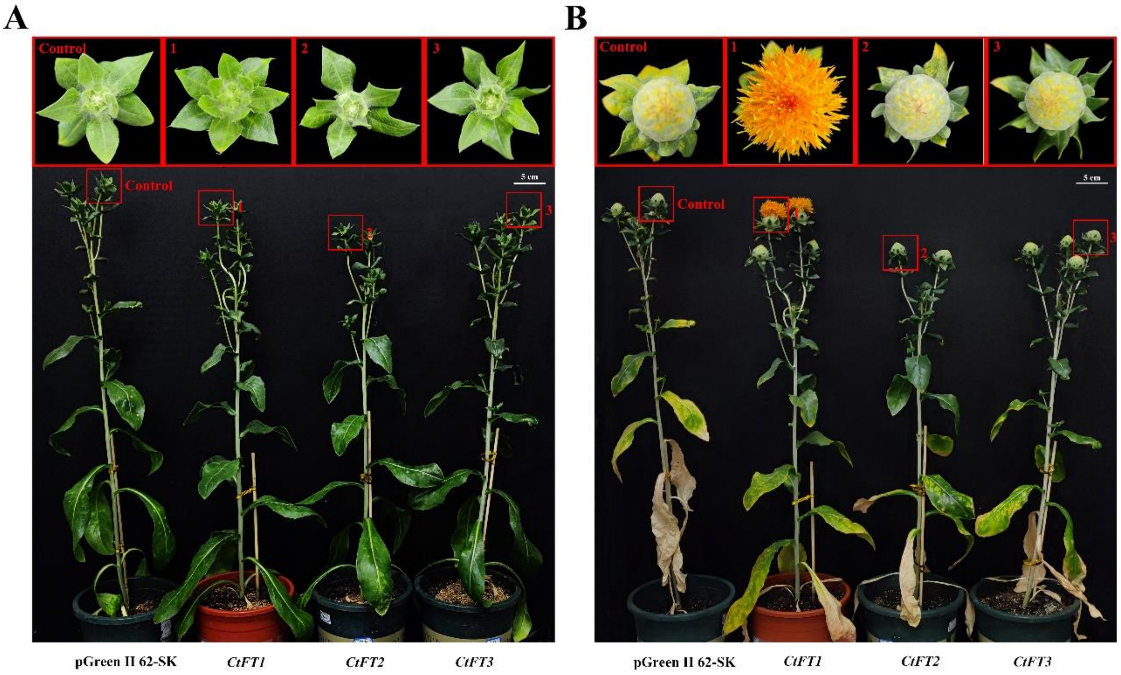

Growth phenotype of transient overexpression of pGreenII62-SK-CtFTs in *Carthamus tinctorius* and injection empty Vector *Carthamus tinctorius*.
A: safflower growth phenotype before injection; B: safflower growth phenotype 20 days after injection.

Figure S7

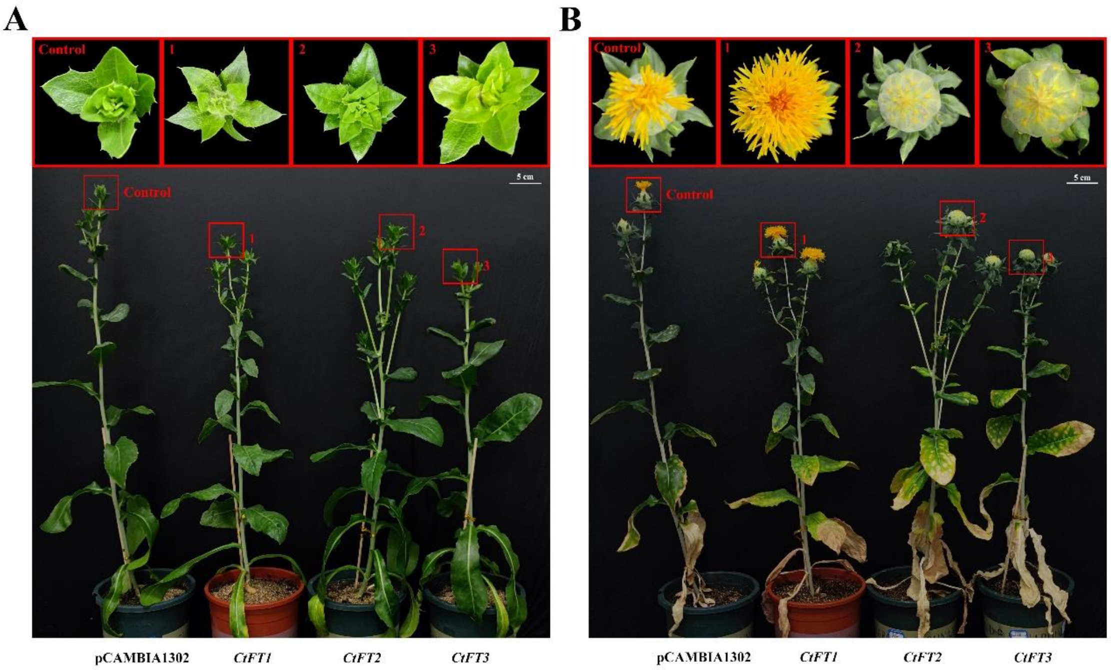

Growth phenotype of transient overexpression of pCAMBIA1302-CtFTs in *Carthamus tinctorius* and injection empty Vector *Carthamus tinctorius*.
A: safflower growth phenotype before injection; B: safflower growth phenotype 20 days after injection
